# Editorial: Neuromorphic engineering for robotics

**DOI:** 10.3389/fnbot.2023.1158988

**Published:** 2023-02-28

**Authors:** Zhenshan Bing, Chenguang Yang, Alois Knoll

**Affiliations:** ^1^Department of Informatics, Technical University of Munich, Munich, Germany; ^2^Bristol Robotics Laboratory, University of the West of England, Bristol, United Kingdom

**Keywords:** robotics, neuromorphic, control, learning, Loihi

Neuromorphic engineering aims to apply insights from neurobiology to develop next-generation artificial intelligence for computation, sensing, and the control of robotic systems. There has been a rapid expansion of neuromorphic engineering technologies for robotics due to several developments. First, the success and limitation of deep neural networks has greatly increased the belief that biological intelligence can further boost the computing performance of artificial intelligence in terms of data, power, and computing efficiency. Second, the emergence of novel neuromorphic hardware and sensors has shown greater application-level performance compared with conventional CPUs and GPUs. Third, the pace of progress in neuroscience has accelerated dramatically in recent years, providing a wealth of new understanding and insights regarding the functioning of brains at the neuron level. Therefore, neuromorphic engineering can represent a fundamental revolution for robotics in many ways. We have published this Research Topic to collect theoretical and experimental results regarding neuromorphic engineering technologies for the design, control, and real-world applications of robotic systems. After carefully and professionally reviewing all submissions, four high-quality manuscripts were accepted. These articles are reviewed below.

Feldotto et al. propose a novel framework to examine the control of biomechanics using physics simulations informed by electromyography (EMG) data. These signals drive a virtual musculoskeletal model in the Neurorobotics Platform (NRP), which is then used to evaluate resulting joint torques. They use their framework to analyze raw EMG data collected during an isometric knee extension study to identify synergies that drive a musculoskeletal lower limb model. The NRP forms a highly modular integrated simulation platform that allows these *in silico* experiments. Their framework allows research of the neurobiomechanical control of muscles during tasks, which would otherwise not be possible. Gu et al. propose a novel American sign language (ASL) translation method based on wearable sensors. By leveraging the initial sensors to capture signs and surface electromyography (EMG) sensors to detect facial expressions, they can extract features from input signals. The encouraging results indicate that the proposed models are suitable for highly accurate sign language translation. With complete motion capture sensors and facial expression recognition methods, the sign language translation system has the potential to recognize more sentences. Ehrlich et al. demonstrate a neuromorphic adaptive control of a wheelchair-mounted robotic arm deployed on Intel's Loihi chip. The proposed controller provides the robotic arm with adaptive signals, guiding its motion while accounting for kinematic changes in real time. They further demonstrate the capacity of the controller to compensate for unexpected inertia-generating payloads using online learning. Akl et al. show how SNNs can be applied to different DRL algorithms, such as the deep Q-network (DQN) and the twin-delayed deep deterministic policy gradient (TD3), for discrete and continuous action space environments, respectively. They show that randomizing the membrane parameters, instead of selecting uniform values for all neurons, has stabilizing effects on the training. They conclude that SNNs can be used for learning complex continuous control problems with state-of-the-art DRL algorithms.

Overall, we hope that this Research Topic can provide some references and novel ideas for the study of neuromorphic robotics.

## Author contributions

All authors listed have made a substantial, direct, and intellectual contribution to the work and approved it for publication.

